# Correction: Cost and statistical efficiency of posture assessment by inclinometry and observation, exemplified by paper mill work

**DOI:** 10.1371/journal.pone.0307247

**Published:** 2024-07-11

**Authors:** Svend Erik Mathiassen, Amanda Waleh Åström, Annika Strömberg, Marina Heiden

In Fig 2, the efficiency ordinate scale in panel B is incorrect. Please see the correct Fig 2 here.

**Fig 2:**
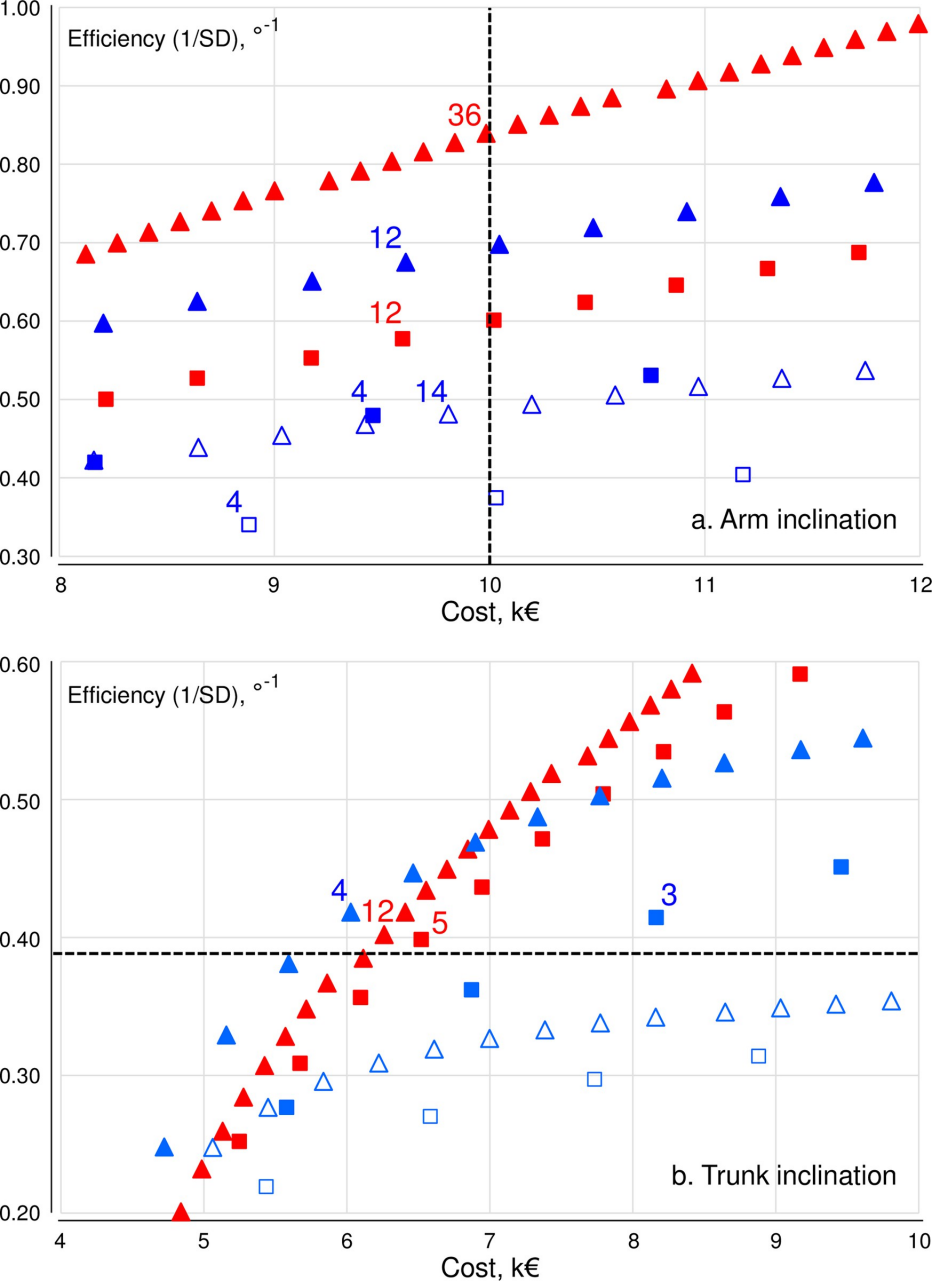


Enlarged sections of Fig 1, corresponding to the examples in the running text of sampling efficiency for different strategies at a specified budget (Fig 2A), and costs for different strategies at a specified efficiency (Fig 2B). Numbers inside the figure show the size of studies complying with the criterion in each of the two cases, i.e. ‘a cost no larger than €10000’ (Fig 2A) and ‘an efficiency corresponding to a 1/SD of at least 0.39°-1’ (Fig 2B). Red and blue symbols: inclinometry and observation; triangles and squares: one shift and three shifts per worker; open and closed symbols: one and three observers.
