# Supplementary material for: Cost and statistical efficiency of posture assessment by inclinometry and observation, exemplified by paper mill work
Source: PLoS One. 2023 Oct 3;18(10):e0292261. doi: 10.1371/journal.pone.0292261 (PMC10547196; doi:10.1371/journal.pone.0292261)
Supplement: S1 File — The document gives a link to a previously published paper in Applied Ergonomics by the current authors, from which the source data for estimation of cost components (Table 1) can be downloaded. (DOCX) [file pone.0292261.s001.docx]

Source data on cost components (cited in Table 1 in the publication) have been published before by the current group of authors in the journal Applied Ergonomics*, with open access.

Data can be downloaded by going to <https://doi.org/10.1016/j.apergo.2018.04.005>, and then to ‘Appendix A. Supplementary data’

* Waleh Åström A, Heiden M, Mathiassen SE, Strömberg A: Uncertainty in monetary cost estimates for assessing working postures using inclinometry, observation or self-report. Appl Ergon 71 (2018): 73–77
