# Supplementary material for: Cost and statistical efficiency of posture assessment by inclinometry and observation, exemplified by paper mill work
Source: PLoS One. 2023 Oct 3;18(10):e0292261. doi: 10.1371/journal.pone.0292261 (PMC10547196; doi:10.1371/journal.pone.0292261)
Supplement: Attachment — Submitted filename: Mathiassen_costefficiency_rebuttalletter_230913.docx [file pone.0292261.s004.docx]

PONE-D-23-09700

Cost and statistical efficiency of posture assessment by inclinometry and observation, exemplified by paper mill work

**Rebuttal letter**

Journal Requirements:

When submitting your revision, we need you to address these additional requirements.

1. Please ensure that your manuscript meets PLOS ONE's style requirements, including those for file naming. The PLOS ONE style templates can be found at

<https://journals.plos.org/plosone/s/file?id=wjVg/PLOSOne_formatting_sample_main_body.pdf> and

<https://journals.plos.org/plosone/s/file?id=ba62/PLOSOne_formatting_sample_title_authors_affiliations.pdf>

We have done our best to follow these templates

2. We noted in your submission details that a portion of your manuscript may have been presented or published elsewhere. [Source data exemplifying the approach described for assessment of cost-efficiency in the present manuscript were taken from (1) two publications by Heiden et al. (references #17 and #23 in the present manuscript), (2) a publication by Waleh-Åström et al. (reference #36). The first author of the present manuscript co-authored all three publications. The present use of data does not constitute dual publication, since the two publications by Heiden et al. were merely descriptive regarding paper mill work, and the publication by Waleh-Åström et al. considered uncertainties in cost assessments. None of the three previous publications addressed cost-efficiency, and data are only used here as an illustration of our suggested approach for how to assess that. Thus, the information communicated by the present manuscript is entirely new.] Please clarify whether this [conference proceeding or publication] was peer-reviewed and formally published. If this work was previously peer-reviewed and published, in the cover letter please provide the reason that this work does not constitute dual publication and should be included in the current manuscript.

We have included the requested information in the enclosed cover letter

3. We note that you have indicated that data from this study are available upon request. PLOS only allows data to be available upon request if there are legal or ethical restrictions on sharing data publicly. For more information on unacceptable data access restrictions, please see <http://journals.plos.org/plosone/s/data-availability#loc-unacceptable-data-access-restrictions>.

In your revised cover letter, please address the following prompts:

    a) If there are ethical or legal restrictions on sharing a de-identified data set, please explain them in detail (e.g., data contain potentially sensitive information, data are owned by a third-party organization, etc.) and who has imposed them (e.g., an ethics committee). Please also provide contact information for a data access committee, ethics committee, or other institutional body to which data requests may be sent.

   b) If there are no restrictions, please upload the minimal anonymized data set necessary to replicate your study findings as either Supporting Information files or to a stable, public repository and provide us with the relevant URLs, DOIs, or accession numbers. For a list of acceptable repositories, please see <http://journals.plos.org/plosone/s/data-availability#loc-recommended-repositories>.

We will update your Data Availability statement on your behalf to reflect the information you provide.

We have described data availability in the enclosed cover letter. Accordingly, we have uploaded supporting information (three files) with a link to the source data for estimation of cost components published in Applied Ergonomics (Waleh-Åström et al. 2018) and two new tables showing data at the individual level for inclinometry and observation, respectively.

4. Please review your reference list to ensure that it is complete and correct. If you have cited papers that have been retracted, please include the rationale for doing so in the manuscript text, or remove these references and replace them with relevant current references. Any changes to the reference list should be mentioned in the rebuttal letter that accompanies your revised manuscript. If you need to cite a retracted article, indicate the article’s retracted status in the References list and also include a citation and full reference for the retraction notice.

We have checked the reference list

Additional Editor Comments:

The review process was lengthy but the outcome is very positive, thank you for your patience.

It was lengthy, indeed… But thank you for having taken it this far 😊

Comments by Reviewer #1

Dear Authors,
I have finished my review on your paper. Indeed, it is quite interesting and well written. I have no major comments and suggestions. Perhaps the Authors would like to discuss in 1-2 sentences how the costs would be affected by running such data collection-processing tasks in other countries and how would new technologies such as AI affect their estimates. Indeed, the use of AI and computer vision to make accurate estimates on postures could be at the beginning but there are many examples of object and instance-based segmentation that could apply to postural checkups.
Best regards and good luck,
S.

We have added that costs are specific to Sweden, but we do not find it justified to discuss any general relationships between costs and efficiency in different countries:
*p.18: While the explicit results of the present study (Fig 1) are specific to the investigated population and measurement strategies, and to the Swedish cost structure, they illustrate some general results that can be expected to apply even to other populations.*

We have also included considerations regarding the future of data collection and costs, in addition to those that were already discussed in the first manuscript. We have specifically mentioned the likely (yet unknown) role of AI and computer vision technology:
*p.20: However, technological developments may, in a more distant future, lead to automated procedures based on Artificial Intelligence (AI), e.g. for determining postures from single camera views in smart phones. This will change the cost-efficiency relationship between inclinometry and observation, and now to the advantage of the latter.*

Comments by Reviewer #2

Reviewer #2: This article is well-written, describing the relative advantages in terms of cost and efficiency of inclinometry and observation (using video technology) considering several levels of variation (number of worker, number of shifts, number of observers). The general premise is interesting and useful. References are excellent and extensive.
This article is worthy of publication with minor revisions. It will be a useful addition to the litterature.

Thank you for your positive review!

It is unclear to me the limitations applying to data availability as stated by the authors. I assume this is because data may be considered proprietary having been the subject of previous publications, however ease of access to this data may yet be a concern for PLOS One rules.

We have uploaded supporting information (three files) with a link to the source data for estimation of cost components published in Applied Ergonomics (Waleh-Åström et al. 2018) and two new tables showing data at the individual level for inclinometry and observation, respectively.

I found the annotation sometimes difficult to follow, despite clear explanations in the written article, since the number of variables was large. Consider using more bullet points (lists) rather than paragraph-based descriptions.

We appreciate that the reviewer considered our explanations to be clear. In the previous manuscript, we already noted the definition of most variables in bullet point lists, but we have now revised the list of measurement units (page 7-8) to also appear in bullet points.

It would be useful to include a second figure that consists of enlarged areas described on pages 12 and 13, with each datapoint explicitly labelled to facilitate direct visual interpretation (not simply based on the text descriptions which are not visible because of scale).

This is an excellent idea, and we have now included two additional figures (fig 2a and 2b), showing enlarged sections of fig 1, so as to clearly illustrate the relationships described on p. 12-13

While I was easily convinced of the validity of equations 2 and 3 (a and b), equations 4 (Q, a and b) remained difficult to follow, causing me to doubt the subsequent interpretations. Consider further explanation in this article around those equations (rather than referencing other works exclusively).

These equations follow standard statistical principles of how to use the results of specified variance component analyses, like those presented in equations 3a and 3b, for estimating the overall variance of a mean exposure. We find that it would require too much information to explain these basic principles, and that it would also lie beyond the purpose of the present study. We have, however, added a sentence explaining that our equations 4a and 4b for estimating statistical performance were developed according to standard statistical principles:
*p.10: Equations 4a and 4b both follow from standard procedures for transforming the variance components in a specific study design (derived using equations 3a and 3b) into the expected statistical performance of a corresponding estimate of a group mean exposure [39].*

… where reference #39 is a statistical textbook (Searle et al. (2006): ‘Variance Components’)

Finally three minor points:
- On p. 13, "assuming normally distributed data" is stated but whether this is a valid assumption is not addressed. Please clarify.

The cited passage in the manuscript refers to an illustrative example of the relative uncertainties of different measurement strategies at the same budget (€ 10000). Thus, normality is not explicitly assumed for the present data, but used as an example so that the reader can get an impression of how different data collection methods compare to one another (at the same cost). We have made that clear by revising the text:
*p.13: If data were, as an example, normally distributed, these SDs…*

- In the Discussion section, observation is assumed to be done manually, whereas recent developments allow angular estimation from a single camera view on a cellphone. Please include this element in your discussions, as you have done for inclinometry technological cost changes.

As commented in the answer to reviewer #1, we have added a sentence in the discussion, in the context of comparing cost-efficiency between inclinometry and observation:
*p.20: However, technological developments may, in a more distant future, lead to automated procedures based on Artificial Intelligence (AI), e.g. for determining postures from single camera views in smart phones. This will change the cost-efficiency relationship between inclinometry and observation, and now to the advantage of the latter.*

- On p. 19, use "custom built" rather than "customary built" for clarity.

We have revised the text as proposed.
